# The Moringin/α-CD Pretreatment Induces Neuroprotection in an In Vitro Model of Alzheimer’s Disease: A Transcriptomic Study

**DOI:** 10.3390/cimb43010017

**Published:** 2021-05-26

**Authors:** Serena Silvestro, Luigi Chiricosta, Agnese Gugliandolo, Renato Iori, Patrick Rollin, Daniele Perenzoni, Fulvio Mattivi, Placido Bramanti, Emanuela Mazzon

**Affiliations:** 1 IRCCS Centro Neurolesi “Bonino-Pulejo”, Via Provinciale Palermo, Contrada Casazza, 98124 Messina, Italy; serena.silvestro@irccsme.it (S.S.); luigi.chiricosta@irccsme.it (L.C.); agnese.gugliandolo@irccsme.it (A.G.); placido.bramanti@irccsme.it (P.B.); 2 Research and Innovation Centre, Department of Food Quality and Nutrition, Fondazione Edmund Mach, 38098 San Michele all’Adige, Italy; renato.iori48@gmail.com (R.I.); daniele.perenzoni@fmach.it (D.P.); fulvio.mattivi@unitn.it (F.M.); 3 Institut de Chimie Organique et Analytique, Université d’Orléans et French Centre National de la Recherche Scientifique (CNRS), ICOA, UMR 7311, BP 6759, F-45067 Orleans, France; patrick.rollin@univ-orleans.fr; 4 Department of Cellular, Computational and Integrative Biology, CIBIO, University of Trento, 38098 San Michele all’Adige, Italy

**Keywords:** moringin/α-CD complex, in vitro study, Alzheimer’s disease, amyloid β-peptide, transcriptomic analysis

## Abstract

Alzheimer’s disease (AD) is a progressive neurodegenerative disorder and represents the most common form of senile dementia. Autophagy and mitophagy are cellular processes that play a key role in the aggregation of β-amyloid (Aβ) and tau phosphorylation. As a consequence, impairment of these processes leads to the progression of AD. Thus, interest is growing in the search for new natural compounds, such as Moringin (MOR), with neuroprotective, anti-amyloidogenic, antioxidative, and anti-inflammatory properties that could be used for AD prevention. However, MOR appears to be poorly soluble and stable in water. To increase its solubility MOR was conjugated with α-cyclodextrin (MOR/α-CD). In this work, it was evaluated if MOR/α-CD pretreatment was able to exert neuroprotective effects in an AD in vitro model through the evaluation of the transcriptional profile by next-generation sequencing (NGS). To induce the AD model, retinoic acid-differentiated SH-SY5Y cells were exposed to Aβ_1-42_. The MOR/α-CD pretreatment reduced the expression of the genes which encode proteins involved in senescence, autophagy, and mitophagy processes. Additionally, MOR/α-CD was able to induce neuronal remodeling modulating the axon guidance, principally downregulating the Slit/Robo signaling pathway. Noteworthy, MOR/α-CD, modulating these important pathways, may induce neuronal protection against Aβ_1-42_ toxicity as demonstrated also by the reduction of cleaved caspase 3. These data indicated that MOR/α-CD could attenuate the progression of the disease and promote neuronal repair.

## 1. Introduction

Neurodegenerative diseases are pathologies characterized by a chronic and selective process of cell death affecting neurons, which occurs mainly in relation to oxidative stress and neuroinflammation. Thanks to their beneficial properties, a large variety of natural compounds showed therapeutic effects against neurodegenerative diseases including Alzheimer’s disease (AD) [1], Parkinson’s disease (PD) [2], and Huntington’s disease (HD) [3]. Medicinal plants such as *Ginseng*, *Ashwagandha*, *Baccopa monnieri*, *Ginkgo biloba*, *Centella asiatica*, and compounds such as flavonoids, celastrol, trehalose, lycopene, sesamol, resveratrol, and curcumin gained a lot of interest for their protective effects in neurodegenerative diseases [4,5]. In particular, the antioxidant [6], anti-inflammatory [7], anti-amyloid [8], anti-apoptotic [9], and cholinergic activities [10] of these compounds may be promising for neurodegenerative disorders. 

Interestingly, Brassicaceae and other species of the order Brassicales, such as Moringaceae have shown health-promoting effects, thanks to their secondary metabolites, the glucosinolates, and their hydrolytic products, the isothiocyanates (ITCs) [11]. *Moringa oleifera* Lam. (Brassicales, family Moringaceae) is a plant used for medicinal purposes due to its high content of ITCs such as moringin (MOR) [12,13]. Due to the sulfur atom in their molecule, ITCs act as antioxidants. Indeed, MOR is a compound capable of exerting protective effects in different models of neurodegenerative disorders [14,15,16]. It was observed that ITCs suppress the activity of acetylcholinesterase, thus prolonging the half-life of acetylcholine, a neurotransmitter whose concentration is usually reduced in patients with AD [17].

AD is the most common cause of senile dementia in the world. The degenerative process progressively affects cells and brain connections, leading to a progressive decline of cognitive functions, the deterioration of personality and relationship life [18]. The hallmarks of AD are the extracellular deposition of amyloid plaques, induced by the aggregation and deposition of the amyloid β-peptide (Aβ), and the formation of intracellular neurofibrillary tangles (NFTs), caused by the hyperphosphorylation of the TAU protein [19,20]. Aβ_1-42_ represents the most abundant form of Aβ in the brain of AD patients [21,22]. Indeed, it is known that Aβ_1-42_ promotes the formation of fibrils contributing to the generation of amyloid plaques [23,24]. The autophagy process plays an important role in Aβ clearance [25,26]. In physiological conditions, the autophagic-lysosome system induces the degradation of Aβ; conversely, in AD alterations of autophagic processes are involved in the accumulation of Aβ [26,27]. Emerging evidence shows that autophagy dysfunctions might be involved in the deposit of Aβ and NTFs in the AD brain [28,29]. Additionally, compromised mitophagy plays a key role in the neurodegenerative process responsible for synaptic dysfunction and neuronal death in AD [30]. Indeed, mitochondrial dysfunction could be an inducer of Aβ aggregation and hyperphosphorylation of TAU [31,32]. However, pre-clinical studies show that Aβ and p-TAU can impair mitochondrial function and integrity, exacerbating AD pathology [33]. Therefore, maintenance of mitochondrial homeostasis is required to prevent AD [34]. Moreover, oxidative stress may accelerate the cellular senescence process, another pathological feature of AD [35]. The reduction of the senescence process has been shown to delay aging-related disorders and alleviate physical dysfunction in old mice [36]. To date, therapeutic interventions are required to improve the pathological features in AD. 

In this context, this study aimed to evaluate the efficacy of the pretreatment with MOR complexed with α-cyclodextrin (α-CD) (MOR/α-CD) against Aβ_1-42_-induced toxicity in an in vitro model of AD performing a Next-Generation Sequencing (NGS)-based gene expression profiling. α-CD is a cyclic hexamer of D-glucose that gives hydrophilic properties to small molecules such as MOR. Indeed MOR appears to be poorly soluble and stable in the water while the complex MOR/α-CD was characterized by an enhanced solubility and stability in water, being α-CD able to form water-soluble complexes with small molecules [37,38,39]. It is important to notice that the MOR/α-CD complex showed similar cytoprotective effects compared to MOR and interestingly, the therapeutic potential of MOR/α-CD seemed to be enhanced [39].

## 2. Materials and Methods

### 2.1. Preparation of the MOR/α-CD Complex

The protocol for the preparation of MOR was developed and initially described by Brunelli et al. [12] and further mentioned in several articles [40,41]. A more recent paper by Mathiron et al. [39] reported the characterization of the MOR/α-CD inclusion complex.

MOR was manufactured via myrosinase-catalyzed hydrolysis of GMG, the glucosinolate precursor isolated from *Moringa oleifera* seeds (cake powder PKM2 provided by Indena India Pvt. Ltd., Bangalore, India), and it was purified by reverse-phase chromatography. The structure was confirmed using AB Sciex Triple Quad 6500+ LC-MS/MS (Applied Biosystems/MDS Sciex, Toronto, ON, Canada) connected with a Waters Acquity HSS T3 column 1.8 μm, 150 mm × 2.1 mm (Milford, MA, USA) kept at 40 °C. 

The mobile phase was composed of eluent A (0.1% formic acid in water) and eluent B (0.1% formic acid in acetonitrile). The flow rate was set to 0.4 mL/min, and the gradient profile was: from 0 to 1 min, isocratic gradient to 98% B; from 1 to 3.50 min, linear gradient to 80% B; from 3.50 to 7 min, isocratic gradient to 80% B; from 7.01 to 9 min, isocratic gradient to 100% and from 9.01 to 12 min, requilibration to the initial conditions of 2% B. The injection volume was 2 μL. 

MOR was analyzed under multiple reaction monitoring (MRM) mode with the following optimized MS conditions. For this ITC, two ion transitions were chosen, one for quantification (quantifier) and one for confirmation (qualifier). In this case, the precursor ion *m/z* was 312.20, and the product ion *m/z* 206.20.

The purity was estimated at >99%, the same degree of purity as that reported by Muller et al. [13]. To obtain the soluble complex MOR/α-CD, 103 mg of solid MOR were added to a solution of 300 mg α-CD (Wacker Chemie AG, München, Germany) in 3.0 mL of water, with a 1:1 M ratio of the two constituents [39]. The resulting aqueous solution was filtered using a 0.45 μm filter, then freeze-dried [42]. Finally, one gram of the prepared complex contained 242.45 mg of MOR.

### 2.2. Cell Culture and Differentiation

The human neuroblastoma cell line SH-SY5Y was obtained from American Type Culture Collection (ATCC) (Manassas, VA, USA). The SH-SY5Y cells were cultured in monolayer using Dulbecco’s Modified Eagle’s Medium/Nutrient Mixture F-12 Ham (DMEM/F12) medium (Sigma-Aldrich, Saint Louis, MO, USA) containing 10% Fetal Bovine Serum (FBS) (Sigma-Aldrich, Saint Louis, MO, USA), glutamine, and penicillin-streptomycin. Cells were grown at 37 °C in a moisturized atmosphere of 5% CO_2_ and 95% air. After culture, SH-SY5Y cells were exposed to 10 µM of retinoic acid (RA) for 5 days, to induce cellular differentiation. 

### 2.3. Cell Treatment with Aβ_1-42_ and MOR/α-CD

Aβ_1-42_ (Sigma-Aldrich, Saint Louis, MO, USA) was dissolved in dimethyl sulfoxide (DMSO) (Fisher Scientific Italia, Rodano, MI, Italy), diluted in phosphate-buffered saline (PBS) (Sigma-Aldrich, Saint Louis, MO, USA), aggregated at 37 °C for 24 h, and added to the medium at the final concentration (final DMSO concentration was <0.1%). Aβ_1-42_ incubated for 24 h at 37 °C forms aggregates, as demonstrated in a previous study [43]. The cells were treated after reaching 80% of confluence in cell culture 6-Well Plates. The cells within passage 10 were seeded with a density of 350,000 cells/well. Cells were pre-treated with MOR/α-CD (0.5 µM) for 24 h. The day after, cells were treated with the medium containing 10 µM of Aβ_1-42_, MOR/α-CD (0.5 µM) alone, or Aβ_1-42_ (10 µM) with MOR/α-CD (0.5 µM) for 24 h. The concentration of Aβ_1-42_ was chosen based on a previous work indicating that it was able to exert cytotoxicity in SH-SY5Y cells [44]. Control cells were incubated with DMEM/F12 medium supplemented with 10% FBS.

### 2.4. Total RNA Extraction and cDNA Library Preparation

The SH-SY5Y’s RNA was obtained using the Maxwell^®^ RSC simplyRNA Cells Kit (Promega, Milan, Italy) according to the manufacturer’s instruction. The library preparation was performed following the TruSeq RNA Exome protocol (Illumina, San Diego, CA, USA) following the instruction, as previously described by Silvestro et al. [45]. In detail, each sample of RNA was fragmented for 8 min at 94 °C. The SuperScript II Reverse Transcriptase (Invitrogen, Milan, Italy) was used to synthetize the first strand of cDNA. After, the second strand was synthesized and purified using AMPure XP beads (Beckman Coulter, Brea, CA, USA). In the next phase, the 3′ ends of the cDNA were adenylated and the index adapter were ligated to the 3′ ends of cDNA. The AMPure XP beads were used to purify the libraries. The cDNA fragments were amplified by PCR and the library were validated through the Agilent Technologies 2100 Bioanalyzer (Santa Clara, CA, USA). The DNA libraries were pooled together using 200 ng for each one and two steps of hybridization followed. The Agilent High Sensitivity Kit Bioanalyzer quantified the final library. Finally, the library was normalized to 12 pM and the sequencing was made in triplicate on the MiSeq Instrument of Illumina (San Diego, CA, USA) in single read mode. 

### 2.5. Transcriptomic Analysis

The fastQC software was used to check the quality of the raw data. Trimmomatic (version 0.38, Usadel Lab, Aachen, Germany) [46] dropped the adapters and low-quality bases and of Spliced Transcripts Alignment to a Reference (STAR) RNA-seq aligner [47] to map the final reads to the mouse reference genome GRCm38. We used the package htseq-count [48] in python to count the transcripts and the package DESeq2 of Bioconductor [49] in R to observe the differential expressed genes between control and Aβ_1-42_ groups as well as Aβ_1-42_ against Aβ_1-42_-MOR/α-CD groups. In detail, the count was modeled following a general linear model and the fold changes for each gene are generated. No filter was selected based on the fold changes of the genes. Based on the modeled counts, the Wald test was applied to check which genes differ in a statistically significant matter. Finally, to drop the number of false positives, all the genes whose q-value obtained after Benjamini–Hochberg correction procedure was higher than 0.05 were rejected. A Volcano Plot was built for each analysis to explore the relationship between the Fold Change a the q-Value for each gene. Then, Reactome was used to enrich the genes for the pathways in which they are included [50]. Again, the False Discovery Rate (FDR) was confirmed with the Benjamini–Hochberg correction procedure. Only the pathways with FDR lower than 0.05 were inspected. The figure was plotted taking advantage of the Protein Data Bank (PDB, rcsb.org) [51] database and in particular of the entry 6Y1A [52].

### 2.6. Protein Extraction and Western Blot Analysis

At the end of the treatment, SH-SY5Y were harvested with trypsin-Ethylenediaminetetraacetic acid (EDTA) and proteins were extracted using the kit NE-PER™ Nuclear and Cytoplasmic Extraction Reagents (Thermo Scientific™, Waltham, MA, USA) following manufacturer protocol. Briefly, after washing with ice-cold PBS, the cells were lysed using ice-cold Cytoplasmatic Extraction Reagent I containing 0.1% of 0.5 M EDTA (Thermo Scientific™, MA, USA) and protease/phosphatase inhibitor cocktail (Thermo Scientific™, MA, USA). The homogenates were chilled on ice for 10 min. Then ice-cold Cytoplasmatic Extraction Reagent II was added, samples were kept on ice for 1 min and centrifuged at 16,000× *g* at 4 °C for 5 min. Then the supernatant (cytosolic extract) was collected. Following, pellets were suspended in Nuclear Extraction Reagent containing 0.1% of 0.5 M EDTA (Thermo Scientific™, USA) and protease/phosphatase inhibitor (Thermo Scientific™, MA, USA), kept on ice for 40 min, and centrifuged at 16,000× *g* at 4 °C for 10 min. The supernatant (nuclear extract) was collected and stored at −80 °C until use. Protein concentrations were determined using a Bio-Rad Protein Assay (Bio-Rad Laboratories) using bovine serum albumin (BSA) as the standard. Twenty-five micrograms of proteins were separated on sodium dodecyl sulfate-polyacrylamide gel electrophoresis (SDS-PAGE) and transferred onto a PVDF transfer membrane (Immobilon-P PVDF, Merck Millipore division of Merck KGaA, Darmstadt, Germany), blocked for 1 h at room temperature, with PBS containing 5% non-fat dried milk. Then, membranes were incubated with selective primary antibodies overnight at 4 °C. 

The following primary antibodies were used: Cleaved-caspase 3 (1:500; Cell Signaling Technology, Danvers, MA, USA) and Caspase 3 (1:500; Cell Signaling Technology, Danvers, MA, USA). The membranes were incubated with secondary antibodies, horseradish peroxidase (HRP)-conjugated anti-rabbit IgG (1:2000; Santa Cruz Biotechnology, Inc., Dallas, TX, USA) for 1 h at room temperature. The expression of Cleaved caspase 3 was normalized on the expression of Caspase 3. The relative expression of protein bands was visualized using an enhanced chemiluminescence system (Luminata Western HRP Substrates, Millipore Corporation, Billerica, MA, USA), and protein bands were obtained and quantified with ChemiDoc™ MP System (Bio-Rad Laboratories S.r.l., Hercules, CA, USA) and analyzed by using the software Image J. All blots are representative of three independent experiments.

### 2.7. Statistical Analysis 

Statistical analysis of Western blot data was performed using GraphPad Prism version 7.0 software (GraphPad Software, La Jolla, CA, USA). The one-way ANOVA test and the Bonferroni post hoc test were carried out for the multiple comparisons. A *p*-value less than or equal to 0.05 was considered statistically significant. The data are expressed by mean ± standard deviation (SD). 

## 3. Results

### 3.1. Experimental Model

Human neuroblastoma SH-SY5Y cells were employed in this experiment because these cells can differentiate and acquire mature neuron-like features. Indeed, in the undifferentiated state SH-SY5Y cells show low levels of neuronal markers. On the contrary, they can acquire a neuron-like phenotype when exposed to differentiation agents, such as RA [53]. To induce neuronal differentiation, cells were exposed to 10 µM RA for 5 days. Subsequently, cells were treated with Aβ_1-42_ to reproduce a model of AD [54,55,56,57]. It is known that Aβ_1-42_ causes neuronal damage and has been widely adopted in several studies to induce AD models also to investigate the protective potential of different compounds [58,59,60]. Previously, it was demonstrated that the treatment of RA-differentiated SH-SY5Y with 10 µM of Aβ_1-42_ for 24 h induced a reduction of cell viability [44]. 24 h prior to incubation with Aβ_1-42_, cells were pretreated with MOR/α-CD, to evaluate the efficacy of this compound. The pretreatment for 24 h was chosen based on previous data indicating that a 24 h-treatment with MOR was not toxic and induced transcriptional changes in genes involved in neuronal processes [61]. Since MOR appears to be poorly soluble and stable in water, it was complexed with α-CD. This new formulation was already evaluated on lipopolysaccharide (LPS)-stimulated RAW 264.7 macrophage cells, showing its therapeutic efficacy for inflammatory diseases [62]. 

### 3.2. Enrichment Analysis

**Figure 1:**
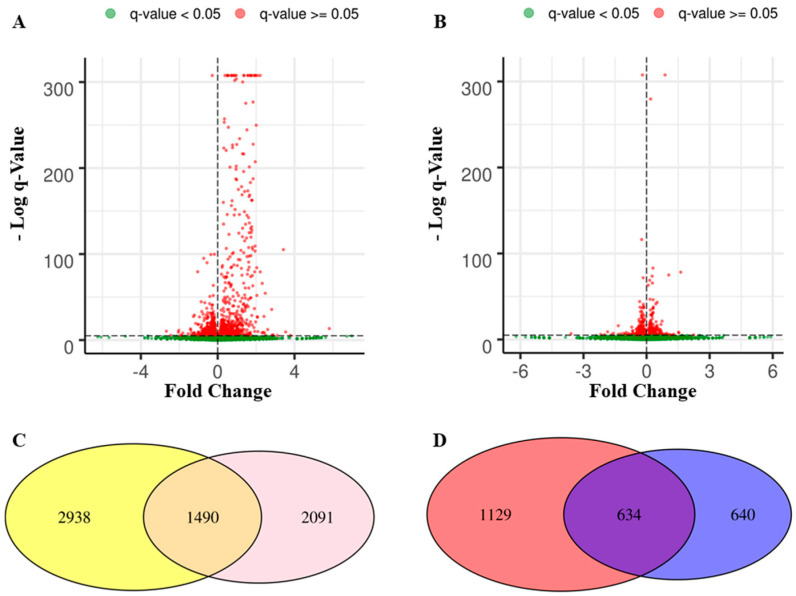
DEGs distribution among control, Aβ_1-42_, and Aβ_1-42_-MOR/α-CD. The distribution of the genes in the comparison of control against Aβ_1-42_ groups is represented in the volcano Plot on the left (**A**) while the panel (**B**) shows the gene distribution for the comparison Aβ_1-42_ against Aβ_1-42_-MOR/α-CD groups. At the bottom, section (**C**) highlighted the DEGs in the two analyses. There are 2938 DEGs in the first analysis that were not deregulated in the second analysis (left yellow panel) and 2091 DEGs in the second analysis that were not included in the first one (right pink panel). In addition, 1490 genes were deregulated in both the analysis. Section (**D**) shows 1129 DEGs upregulated in the first analysis that were not downregulated in the second one (**left red panel**) and the 640 DEGs downregulated in the second analysis that were not upregulated in the first one (**right blue panel**). The 634 DEGs simultaneously upregulated in the first analysis and downregulated in the second one (central panel) are the focus of this study.

In the first analysis, control against Aβ_1-42_ groups revealed 2031 downregulated and 2397 upregulated genes that were distributed as shown in Figure 1A. On the other hand, in the second analysis, the comparison of Aβ_1-42_ against Aβ_1-42_-MOR/α-CD groups showed 1908 downregulated and 1673 upregulated genes (Figure 1B). The Venn diagram in Figure 1C shows how the differentially expressed genes (DEGs) were distributed between the two analyses. In detail, 2938 genes were deregulated in the first analysis but they were not in the second one (left yellow panel). Conversely, the right pink panel shows that 2091 genes were deregulated only in the second analysis. Moreover, 1490 genes were differentially expressed and shared between the two analyses, for this reason, they are represented in the intersection. From this point, the analysis was focused on the genes upregulated in the first analysis and downregulated in the second one. Thus, the Venn diagram in Figure 1D shows the 1129 genes upregulated in the first set that were not downregulated in the second analysis (left red panel) and the 640 genes downregulated in the second analysis that were not upregulated in the first one (right blue panel). The purple intersection highlighted 634 DEGs that were upregulated in the first analysis and downregulated in the second one. These 634 DEGs were further analyzed using the pathway database Reactome. 

**Figure 2:**
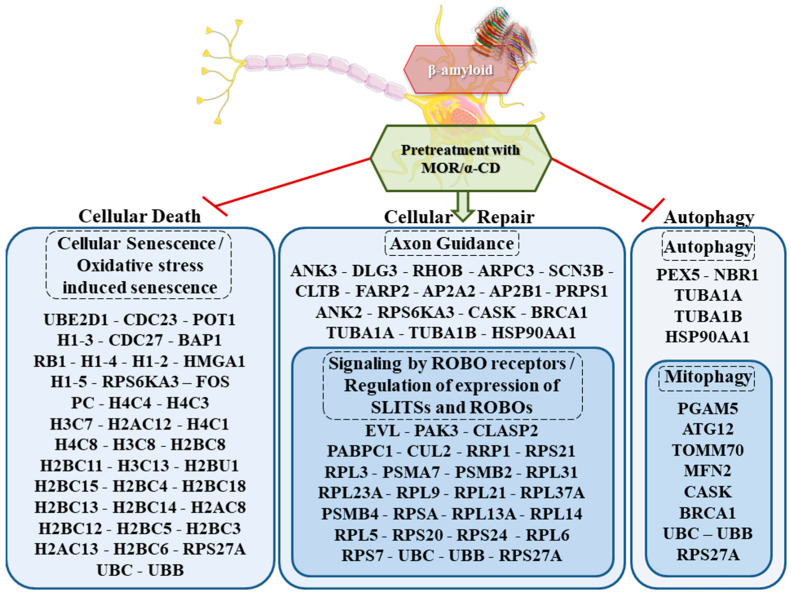
Biological clusters of DEGs activated by MOR/α-CD action. The analysis of differential expressed genes upregulated between control and Aβ_1-42_ groups revealed 97 genes downregulated in the analysis of Aβ_1-42_ against Aβ_1-42_-MOR/α-CD and they are represented in the Figure. They are enriched in Reactome for the pathways "Oxidative stress-induced senescence" and “Senescence” represented in the “Cellular Death” cluster, for “Autophagy” and “Mitophagy” in the “Autophagy” one. The downregulation of the genes in these pathways highlights the inhibitor effects of MOR/α-CD. On the other hand, the downregulation of the genes in the “Cellular Repair” cluster with “Regulation of expression of SLITs and ROBOs”, “Signaling by ROBO receptors” and “Axon Guidance” showed the increased effect of synaptic plasticity. The β-amyloid structure (6Y1A) was retrieved by PDB. The figure was drawn using the vector image bank of Servier Medical Art by Servier (smart.servier.com) (accessed on April 2021). Licensed under a Creative Commons Attribution 3.0 Unported License (creativecommons.org/licenses/ by/3.0/) (accessed on April 2021).

“Cellular Death”, “Cellular Repair” and “Autophagy” were the main cluster identified. In detail, as represented in Figure 2, 39 genes were enriched for the pathways “Senescence” and “Oxidative stress induced-senescence”, 44 genes for “Axon Guidance”, “Signaling by ROBO receptors” and “Regulation of expression of SLITs and ROBOs” while 14 genes for “Autophagy” and “Mitophagy”. In Table 1 *UBB*, *UBC* and *RPS27A* were included, three ubiquitins that are deregulated in all the categories. In addition, Table 2 shows 36 genes deregulated in “Cellular Death” among which *RPS6KA3* was deregulated also in “Cellular Repair”. The 41 genes in Table 3 were included in the category “Cellular Repair” and *RPS6KA3* was shared with “Cellular Death”, while *CASK*, *BRCA1*, *TUBA1A*, *TUBA1B*, and *HSP90AA1* were in common with “Autophagy”. The 11 genes of “Autophagy” are shown in Table 4.

**Figure 3:**
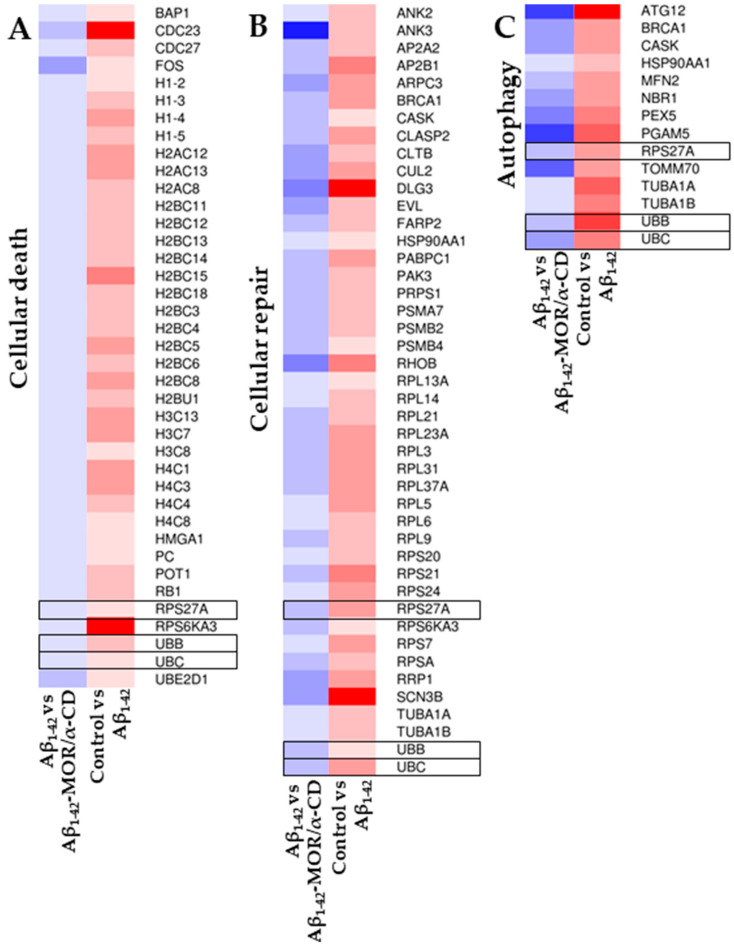
Heatmaps of the inspected DEGs. The heatmaps represent the DEGs found in Cellular Death” (**A**), “Cellular Repair” (**B**), and “Autophagy” (**C**) categories. The color scale goes from blue for the downregulated to red for the upregulated genes. By construction, all the genes in the left panel (Aβ_1-42_ against Aβ_1-42_-MOR/α-CD groups) are downregulated whereas all the genes in the right panel (control and Aβ_1-42_ groups) are upregulated. In the black frame were highlighted the ubiquitins *UBB*, *UBC*, and *RSP27A* that are deregulated in all the categories.

To better visualize the changes in the gene expression values, a heatmap was represented in Figure 3. It highlighted the differences in the expression level of the aforementioned DEGs in the three different categories “Cellular Death”, “Cellular Repair” and “Autophagy”, with a color scale that goes from blue, for the downregulated genes, to red, for the upregulated ones. The heatmap clearly highlighted that the considered DEGs were upregulated in the comparison between control and Aβ_1-42_ groups, while the same genes were downregulated in the comparison Aβ_1-42_ against Aβ_1-42_-MOR/α-CD groups. The genes *UBB*, *UBC*, and *RSP27A* were highlighted with a black frame to evidence that they were deregulated in all the categories.

### 3.3. Evaluation of Cleaved Caspase 3 by Western Blot Analysis

**Figure 4:**
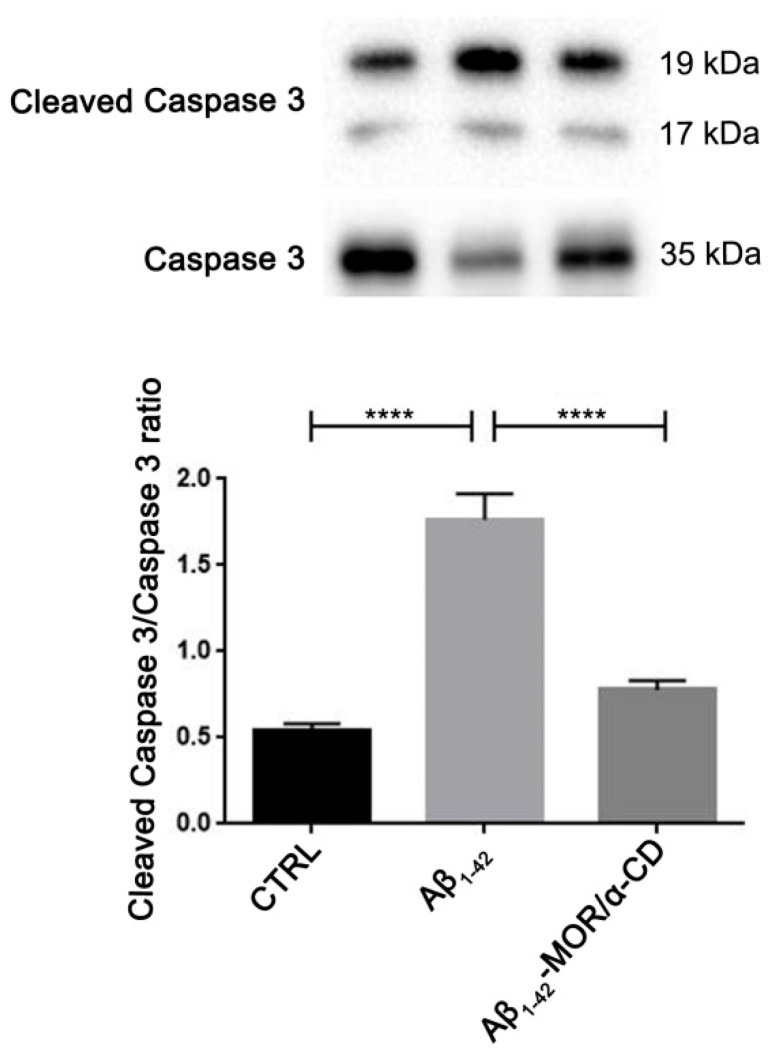
Western Blot analysis for Cleaved caspase 3. Aβ_1-42_ treatment increased protein levels of Cleaved caspase 3, indicating the induction of apoptosis. MOR/α-CD pretreatment reduced cleaved caspase 3 levels, indicating that it can exert a protective action against Aβ_1-42_-induced apoptosis. **** *p* < 0.0001.

To verify the protective role exerted by MOR/α-CD pretreatment, the protein levels of Cleaved caspase 3 were evaluated with western blot analysis. We observed higher levels of cleaved caspase 3 in RA-differentiated SH-SY5Y treated with Aβ_1-42_ (Figure 4), indicating the induction of apoptosis induced by Aβ_1-42_. Interestingly, MOR/α-CD pretreatment reduced cleaved caspase 3 protein levels, suggesting that MOR/α-CD pretreatment was able to exert a protective role against Aβ_1-42_-induced cell death. It is important to notice that MOR/α-CD pretreatment reduced cleaved caspase 3 to levels similar to those of control cells.

## 4. Discussion

Recently, evidence about the neuroprotective effects of ITCs have been obtained in in vitro and in vivo models of neurodegeneration [14,63,64,65,66,67]. 

In this experiment, RA-differentiated SH-SY5Y cells were exposed to Aβ_1-42_ to induce a model of AD, to investigate the efficacy of MOR/α-CD pretreatment against Aβ_1-42_-induced toxicity. MOR/α-CD treatment did not induce cytotoxic effects, compared to untreated cells, and affected the expression of many genes modulated also in the Aβ_1-42_ group. Previously, SH-SY5Y cells were exposed to different concentrations of MOR for 24 h, to assess its cytotoxic effect. It was demonstrated that concentrations of MOR ranging from 1.64 to 8.2 µM, did not cause SH-SY5Y cell death or increase lactate dehydrogenase (LDH) release [68]. Additionally, it was already shown that MOR at a concentration of 0.5 µM did not induce cell death, but conversely increased cell proliferation [61,69]. In this study, the dose of 0.5 µM of MOR/α-CD and the 24 h pretreatment were chosen based on a previous study demonstrating that this dose at this timepoint influenced the expression of genes involved in neuronal differentiation, while higher doses did not condition the fold change of the genes [61]. Additionally, previously it was already observed that MOR treatment alone downregulated most of the genes involved in mitophagy [70].

This work aimed to evaluate the changes in the transcriptional profile induced by pretreatment with MOR/α-CD in SH-SY5Y cells treated with Aβ_1-42_. The NGS-based gene expression profile was performed to assess the modifications in the gene expression of the transcripts involved in pathways altered in AD.

**Figure 5:**
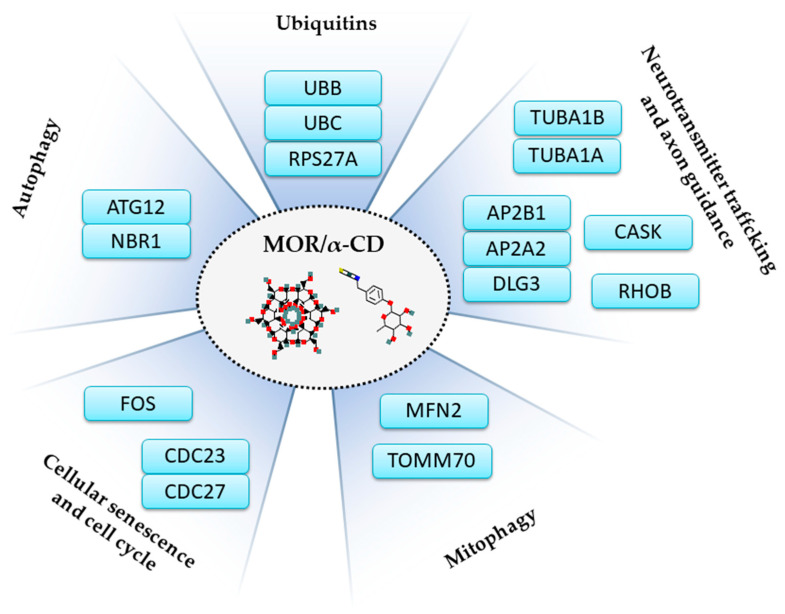
Illustration of the mechanisms operated by MOR/α-CD found in the analysis. MOR/α-CD pretreatment specifically downregulates genes involved in autophagy (*ATG12, NBR1*), mitophagy (*MFN2, TOMM70*), ubiquitins (*UBB, UBC, RPS27A*), cellular senescence and cell cycle (*CDC23, CDC27, FOS*), neurotransmitter trafficking, and axon guidance (*CASK, AP2A2, AP2B1, DLG3, RHOB, TUBA1A, TUBA1B*). The images of MOR (pubchem.ncbi.nlm.nih.gov/compound/14865502#section=2D-Structure) (accessed on May 2021). and α-CD (pubchem.ncbi.nlm.nih.gov/compound/444913#section=2D-Structure) (accessed on May 2021) were retrieved by PubChem.

In this transcriptomic analysis, pretreatment with MOR/α-CD showed the downregulation of three main gene clusters, which are “Cellular Death”, “Autophagy”, and “Cellular Repair”, represented in Figure 1. Among these genes, *UBC*, *UBB*, and *RPS27A*, listed in Table 1, are commonly expressed in all of the three gene clusters and they encode for the ubiquitins, important regulators of most cellular protein networks (Figure 5). Impairment of the ubiquitin-proteasome system exerts pleiotropic effects in the neurodegenerative brain contributing to neurotoxicity, synaptic dysfunction, and consequently cell death [71]. Therefore, compounds that target specific ubiquitin-proteasome system components in the nervous system may represent a therapeutic approach [72].

Pretreatment with MOR/α-CD downregulated a group of genes, listed in Table 2, that are enriched in “Cellular Senescence” and “Oxidative Stress Induced Senescence”.

Senescent cells are involved in neurodegeneration, promote insoluble tau aggregates, and cognitive impairments. Conversely, treatment with senolytic compounds reduced the overexpression of genes associated with senescence and the progression of the tau-mediated disease [73]. In this analysis, MOR/α-CD pretreatment was able to reduce the expression of *FOS*. Interestingly, *FOS* is a gene specific of senescence that encodes for c-Fos, a nuclear protein involved in the regulation of cell proliferation, differentiation, and death [74]. It was observed that Aβ-induced O-GlcNAcylation promotes the transcriptional activity of c-Fos responsible for neuronal cell death through the enhancement of the level of the apoptotic protein Bim. Moreover, pretreatment with MOR/α-CD reduced *CDC23* and *CDC27* expression, two genes that encode for components of the anaphase-promoting complex/cyclosome (APC/C), involved in the control of the progression through mitosis and the G1 phase of the cell cycle [75]. Therefore, the downregulation of *FOS*, *CDC23*, and *CDC27* could be an important mechanism used by MOR/α-CD to regulate cell cycle progression and senescence, protecting the cell from Aβ-induced apoptosis and attenuating the progression of the disease. In this regard, Western Blot analysis showed that MOR/α-CD pretreatment could decrease cleaved caspase 3 protein levels compared to the Aβ_1-42_ group, indicating a reduction of Aβ_1-42_-induced apoptosis (Figure 4). 

Emergent evidence showed that the dysfunction of autophagy is involved in the pathophysiology of AD [27,76]. In AD brains, autophagosomes may represent a reservoir of Aβ; thus, an increase of new autophagosomes could induce an increase in Aβ production, accumulation, and as a consequence, its toxicity [27]. Indeed, altered functions of specific autophagic genes were observed in several pre-clinical studies, and in human brain samples of AD patients [29,77,78]. In this context, the results of this study showed that MOR/α-CD-pretreatment downregulated several genes involved in the “Autophagy” process, listed in Table 3. Among them, *ATG12* is encoded for Autophagy Related 12, a ubiquitin-like protein involved in autophagic vesicle formation [44]. Instead, *NBR1* encodes for Autophagy Cargo Receptor that plays a role as a receptor for selective autophagosomal degradation of ubiquitinated targets [79]. Our results may indicate that MOR/α-CD pretreatment, downregulating these genes, could restore the autophagic pathway. Therefore, autophagy could be a promising therapeutic target used by MOR/α-CD to improve the pathogenesis of AD.

Interestingly, a group of genes represented in the “Autophagy” group (Table 4) is also implicated in the “Mitophagy” sub-pathway. Mitophagy is a biological process responsible for the removal of aberrant and aged mitochondria [80]. Altered mitophagy processes appear responsible for mitochondrial dysfunction, which induces oxidative stress, an important event underlying AD [81,82]. In this study, pretreatment with MOR/α-CD downregulated these genes including *TOMM70*, *MFN2*, *PGAM5*, *ATG12*, *PEX5*, *NBR1*, *CASK*, and *BRCA1*. In line with these results, previously it was demonstrated that pretreatment of human periodontal ligament stem cells with MOR induced the downregulation of genes involved in mitophagy [70]. Among these genes, *TOMM70*, *MFN2*, and *ATG1* are involved in the PINK1-PRKN mediated mitophagy, which is a molecular axis regulated by two principal genes associated with mitochondrial kinase PTEN-induced kinase 1 (PINK1) and ubiquitin ligase Parkin (PRKN). This molecular axis plays an important role in recognizing damaged mitochondria and in label them for autophagic degradation [83]. In normal conditions, PINK1 acts as a stress sensor involved in the detection of mitochondrial quality. Conversely, changes in the PINK1 expression could be related to neurodegenerative disorders such as AD and PD [84,85]. *TOMM70* encode for Translocase of Outer Mitochondrial Membrane 70, a subunit of the outer mitochondrial membrane translocase important as a receptor of hydrophobic pre-proteins targeted to mitochondria [86]. Recently it was demonstrated that the development of inhibitors of molecular interaction between Hsp70/Hsp90 and TOMM70 results in a reduction in mitochondria-associated amyloid precursor protein (APP) and protects SH-SY5Y cells from the toxic effect of Aβ_1-42_ exposure [87]. *MFN2* encode for Mitofusin 2, homologous outer membrane large GTPases that govern mitochondrial fusion and Parkin ubiquitination substrates [88], mediating signaling activity of the PINK1-Parkin. Thus, these data suggest that mitochondrial dysfunctions induced by oxidative stress can be inhibited by MOR/α-CD pretreatment, through downregulation of the genes involved in mitophagy.

Noteworthy, *CASK*, *BRCA1*, *TUBA1A*, *TUBA1B*, and *HSP90AA1* represented in the “Autophagy” cluster, are commonly expressed also in the “Cellular repair” group. *CASK* is a pleiotropic gene that encodes a calcium/calmodulin-dependent serine protein kinase (CASK). This protein plays a key role in synaptic transmembrane protein anchoring and ion channel trafficking [89]. In neurons, CASK is involved in pre and postsynaptic signaling [90]. Nevertheless, it was demonstrated that interactions between amyloid precursor protein-like (APPL) and scaffolding proteins such as CASK could also be involved in recruiting α-secretase at the synaptic site as well as generating sAPPa [91]. The *BRCA1* gene encodes for a protein involved in transcription, repair of double-strand breaks, and recombination [92]. In AD brains a depletion of BRCA1, likely due to Aβ_1-42_, enhanced the cognitive deficits [93]. On the other hand, it was demonstrated that upregulation of the BRCA1 protein could both influence Presenilin 1 turnover, leading to Aβ_1-42_ pathology, and promote cell cycle re-entry-driven cell death of postmitotic neurons in AD [94]. *TUBA1A* and *TUBA1B* encode for alpha and beta tubulins, the major components of microtubules. The microtubule cytoskeleton forms the tracks on which motor proteins bind to facilitate intracellular transport of mitochondria and lysosomes [95]. In adult neurons, microtubules play a key role in synapse plasticity, axon growth, and guidance [96]. However, how microtubules are regulated to support distinct roles in different compartments of the neuron is still unknown. *HSP90AA1* encode for Heat Shock Protein 90 Alpha Family Class A Member 1, belongs to the HSP90 family, one of the most important heat shock proteins. This protein is involved in mitochondrial import, delivers pre-proteins to the mitochondrial receptor TOMM70 [97]. Moreover, it has been reported that the Hsp90α overexpression in the stress response, may be useful in addressing neurological pathologies related to protein aggregation [98]. MOR/α-CD-induced downregulation of these genes could protect the neuronal cells from damage due to Aβ_1-42_ toxicity, could regulate the mitochondrial import, and play a key role in synaptic plasticity, axon growth, and guidance.

*AP2A2*, *AP2B1*, and *DLG3* encode for proteins located in the postsynaptic neurons, involved in the maturation of the synapses and the neurotransmitters trafficking. These genes, represented in the "Cellular Repair" group (Table 3), are enriched in the "Axon Guidance" pathway. *AP2A2* and *AP2B1* encode for subunits of the adaptor protein complex 2 (AP-2). The AP-2 complex reduces the excitatory stimuli by endocytosis of α-amino-3-hydroxy-5-methyl-4-isoxazolepropionic acid receptors (AMPARs) [99]. *DLG3* encodes for the protein Disks Large homolog 3 that stabilizes N-methyl-D-aspartate receptors (NMDARs) [100]. In this study, pretreatment with MOR/α-CD downregulated these genes which, conversely, were upregulated in the Aβ_1-42_ group. Besides downregulating these genes, the pretreatment also reduced *RHOB*, encoded for Ras Homolog Family Member B (RHOB), a Rho family GTPase. These proteins are involved in several functions in cells and are expressed in the adult brain in areas of high synaptic plasticity [101]. The pathways involved in axon guidance are modulated by several classic guidance families, such as Netrins, Slits, Semaphorins, and Ephrins, Robo, Neuropilins/Plexins, and Ephs [102]. Among these, the Slit family proteins (Slit) and Roundabout (Robo) receptors are evolutionary conserved chemorepulsive axon guidance molecules primarily known for their function in regulating midline major forebrain cortical axonal tracts [103,104]. However, it was demonstrated that SLIT reduced dopaminergic neuronal extension and neurite growth, suppressing neuroplasticity [105]. This process was also demonstrated in neurites of stem cell-derived neurons [106]. It was also observed that downregulation of RhoA, a negative regulator involved in the SLIT/ROBO pathway stimulated neuronal growth [107]. Therefore, the downregulation of genes involved in the Slit/Robo pathway induced by MOR/α-CD could promote neuronal plasticity. In this regard, it was observed that the attenuation of Robo signaling would drive the expansion and increased the complexity of the mammalian cerebral cortex [108]. Thus, the regulation of the genes involved in the synapse maturation and neurotransmitter trafficking may be a mechanism used by MOR/α-CD to regulate synaptic remodeling, altered in AD.

Considering that there are no drugs capable of stopping or reversing the disease, the need to find prevention strategies is growing. Based on preclinical studies, nutraceutical compounds, such as MOR, could potentially be effective in achieving excellent results on cognitive performance in patients with AD [109]. The MOR/α-CD pretreatment may be relevant for the observed effects, as early and continuous intake may improve efficacy over time. Natural compounds are involved in the regulation of mitochondrial stress, free radical scavenging systems, and apoptotic factors [110]. The progression of AD is also accelerated by oxidative stress and inflammation contributing to neurodegeneration. Therefore, early prevention and management might be used as a potential treatment for reducing symptoms of AD [111].

These data indicated that that the treatment of SH-SY5Y with Aβ_1-42_ altered different processes such as autophagy, mitophagy, cell senescence, ubiquitin system, and axon guidance. Indeed, these processes are known to be altered in AD. MOR/α-CD can modulate the expression of genes encoding for proteins involved in these processes (Figure 5) exerting neuroprotective effects and reducing cell death. Then, MOR/α-CD could represent a promising approach against AD.

## 5. Conclusions

MOR complexed with α-CD could be an interesting approach to improve and overcome the poor bioavailability of the phytocompound. Pretreatment with MOR/α-CD could induce neuronal protection against Aβ_1-42_-toxicity, downregulating genes involved in cellular senescence. Furthermore, autophagy and mitophagy, which represent major pathways for the elimination of aggregated proteins and organelles, could be emerging and promising therapeutic targets modulated by MOR/α-CD to ameliorate AD. Additionally, MOR/α-CD, through the downregulation of some genes involved in the Axon Guidance process, could regulate synaptic plasticity. Therefore, it will be necessary to translate these results in in vivo experimental models, to assess the molecular mechanism exerted by MOR/α-CD. However, these data encourage future studies to investigate the use of MOR/α-CD supplementation in patients with AD.

## Figures and Tables

**Table 1:** Differentially expressed genes exclusively enriched in “Cellular Death”, “Cellular Repair” and “Autophagy” groups simultaneously.

Gene	Aβ_1-42_	Aβ_1-42_-MOR/α-CD	Fold Change	*q*-Value
*UBC*	28,343.77	25,319.99	−0.16	1.77 × 10^−72^
*UBB*	18,164.72	16,320.22	−0.15	1.08 × 10^−41^
*RPS27A*	5099.52	4736.33	−0.11	6.81 × 10^−06^

Fold change for each gene obtained from differentially expressed gene analysis between Aβ_1-42_ against Aβ_1-42_- MOR/α-CD-treated groups.

**Table 2:** Differentially expressed genes exclusively enriched in “Cellular Death” group.

Gene	Aβ_1-42_	Aβ_1-42_-MOR/α-CD	Fold Change	*q*-Value
*FOS*	165.57	65.15	−1.35	9.2 × 10^−17^
*UBE2D1*	195.07	129.26	−0.59	9.17 × 10^−06^
*CDC23*	457.71	334.18	−0.45	4.25 × 10^−08^
*H4C4*	1214.22	933.18	−0.38	2.78 × 10^−15^
*POT1*	391.1	299.5	−0.38	2.36 × 10^−05^
*H1-3*	1534.9	1236.89	−0.31	2.3 × 10^−13^
*H4C3*	885.92	718.8	−0.3	2.08 × 10^−07^
*H3C7*	4321.13	3535.17	−0.29	4.85 × 10^−33^
*PC*	224.57	184.96	−0.28	2.91 × 10^−2^
*H2AC12*	2469.35	2042.92	−0.27	7.77 × 10^−17^
*CDC27*	932.55	782.91	−0.25	9.82 × 10^−06^
*H4C1*	528.13	448.73	−0.24	3.26 × 10^−3^
*H4C8*	3100.25	2646.12	−0.23	5.12 × 10^−15^
*BAP1*	960.14	835.45	−0.2	4.97 × 10^−4^
*RB1*	1472.09	1294.69	−0.19	4.42 × 10^−05^
*H3C8*	2054.46	1795.96	−0.19	2.05 × 10^−07^
*H2BC8*	4683.68	4155.19	−0.17	2.98 × 10^−13^
*RPS6KA3*	659.45	593.75	−0.15	3.95 × 10^−2^
*H1-4*	2895.66	2604.09	−0.15	1.12 × 10^−06^
*H2BC11*	2434.14	2212.11	−0.14	8.72 × 10^−05^
*H3C13*	1587.24	1438.66	−0.14	1.59 × 10^−3^
*H2BU1*	1345.53	1231.64	−0.13	1.04 × 10^−2^
*H2BC15*	2958.46	2709.18	−0.13	6.31 × 10^−05^
*H2BC4*	7610.74	7008.34	−0.12	1.68 × 10^−10^
*H1-2*	3724.49	3435.34	−0.12	3.3 × 10^−05^
*H2BC18*	18,225.62	16,846.71	−0.11	8.37 × 10^−23^
*HMGA1*	2043.04	1887.39	−0.11	4.03 × 10^−3^
*H2BC13*	5959.75	5545.51	−0.1	1.77 × 10^−06^
*H2BC14*	6351.8	5937.49	−0.1	4.12 × 10^−06^
*H1-5*	3449.48	3224.11	−0.1	1.2 × 10^−3^
*H2AC8*	4656.08	4368.52	−0.09	3.18 × 10^−4^
*H2BC12*	11,248.63	10,570.84	−0.09	5.96 × 10^−09^
*H2BC5*	6795.23	6390.42	−0.09	1.62 × 10^−05^
*H2BC3*	2053.51	1931.52	−0.09	3.08 × 10^−2^
*H2AC13*	2836.66	2695.52	−0.07	3.45 × 10^−2^
*H2BC6*	7681.16	7295.23	−0.07	1.57 × 10^−4^

Fold change for each gene obtained from differentially expressed gene analysis between Aβ_1-42_ against Aβ_1-42_-MOR/α-CD-treated groups.

**Table 3:** Differentially expressed genes exclusively enriched in “Cellular Repair” group.

Gene	Aβ_1-42_	Aβ_1-42_-MOR/α-CD	Fold Change	*q*-Value
*ANK3*	59.95	28.37	−1.08	8.64 × 10^−^^05^
*DLG3*	185.56	121.9	−0.61	1.08 × 10^−05^
*RHOB*	77.08	51.49	−0.58	1.08 × 10^−2^
*CUL2*	422.5	312.11	−0.44	4.79 × 10^−07^
*ARPC3*	2117.27	1650.94	−0.36	4.29 × 10^−24^
*SCN3B*	233.14	183.9	−0.34	5.65 × 10^−3^
*CLTB*	243.6	196.52	−0.31	1.06 × 10^−2^
*EVL*	728.91	590.6	−0.3	2.83 × 10^−06^
*RRP1*	271.2	221.74	−0.29	1.13 × 10^−2^
*RPS21*	587.13	497.07	−0.24	1.38 × 10^−3^
*RPL3*	7708.75	6583.78	−0.23	2.44 × 10^−37^
*CASK*	1451.16	1244.25	−0.22	7.86 × 10^−07^
*FARP2*	348.28	301.6	−0.21	4.27 × 10^−2^
*PAK3*	490.06	427.71	−0.2	2.04 × 10^−2^
*PSMA7*	690.85	600.05	−0.2	3.4 × 10^−3^
*PSMB2*	612.82	538.05	−0.19	1.22 × 10^−2^
*RPL31*	4263.08	3770.57	−0.18	1.09 × 10^−12^
*BRCA1*	1762.33	1571.07	−0.17	6.32 × 10^−05^
*AP2A2*	916.37	814.43	−0.17	4.66 × 10^−3^
*AP2B1*	2839.52	2542.09	−0.16	4.64 × 10^−07^
*RPS6KA3*	659.45	593.75	−0.15	3.95 × 10^−2^
*PRPS1*	676.57	609.51	−0.15	3.77 × 10^−2^
*CLASP2*	3262.97	2984.51	−0.13	1.66 × 10^−05^
*RPL23A*	19,377.03	17,521.38	−0.15	2.07 × 10^−39^
*RPL9*	20,697.82	18,816.07	−0.14	7.18 × 10^−38^
*RPL21*	7862.91	7213.26	−0.12	8.29 × 10^−12^
*RPL37A*	5608.61	5145.13	−0.12	1.69 × 10^−08^
*PSMB4*	1606.27	1481.75	−0.12	1.05 × 10^−2^
*RPSA*	23,320.38	21,581.99	−0.11	2.03 × 10^−28^
*PABPC1*	12,540.88	11,712.1	−0.1	6.05 × 10^−12^
*RPL13A*	18,196.12	17,153.57	−0.09	6.00 × 10^−13^
*RPL14*	7532.71	7059.84	−0.09	1.24 × 10^−06^
*RPL5*	6921.8	6569.07	−0.08	2.99 × 10^−4^
*RPS20*	5426.86	5147.23	−0.08	1.44 × 10^−3^
*RPS24*	16,100.74	15,214.69	−0.08	1.30 × 10^−10^
*RPL6*	5468.73	5242.86	−0.06	1.3 × 10^−2^
*ANK2*	8085.58	7733.45	−0.06	9.88 × 10^−4^
*TUBA1A*	39,386.86	38,099.78	−0.05	4.31 × 10^−09^
*TUBA1B*	28,968.96	28,183.64	−0.04	7.27 × 10^−05^
*RPS7*	12,767.35	12,416.19	−0.04	1.16 × 10^−2^
*HSP90AA1*	17,476.72	17,009.6	−0.04	3.48 × 10^−3^

Fold change for each gene obtained from differentially expressed gene analysis between Aβ_1-42_ against Aβ_1-42_- MOR/α-CD-treated groups.

**Table 4:** Differentially expressed genes exclusively enriched in “Autophagy” group.

Gene	Aβ_1-42_	Aβ_1-42_-MOR/α-CD	Fold Change	*q*-Value
*PGAM5*	144.64	101.94	−0.5	1.61 × 10^−3^
*ATG12*	150.35	107.19	−0.49	1.84 × 10^−3^
*TOMM70*	632.8	492.86	−0.36	2.17 × 10^−07^
*PEX5*	764.12	622.12	−0.3	2.69 × 10^−06^
*CASK*	1451.16	1244.25	−0.22	7.86 × 10^−07^
*NBR1*	601.4	520.19	−0.21	5.17 × 10^−3^
*BRCA1*	1762.33	1571.07	−0.17	6.32 × 10^−05^
*MFN2*	1434.98	1339.88	−0.1	4.58 × 10^−2^
*TUBA1A*	39,386.86	38,099.78	−0.05	4.31 × 10^−09^
*TUBA1B*	28,968.96	28,183.64	−0.04	7.27 × 10^−05^
*HSP90AA1*	17,476.72	17,009.6	−0.04	3.48 × 10^−3^

Fold change for each gene obtained from differentially expressed gene analysis between Aβ_1-42_ against Aβ_1-42_- MOR/α-CD-treated groups.

## Data Availability

The data presented in this study are openly available in the NCBI Sequence Read Archive at BioProject accession number PRJNA719033.
